# Oncogenic Ras suppresses ING4-TDG-Fas axis to promote apoptosis resistance

**DOI:** 10.18632/oncotarget.6015

**Published:** 2015-10-24

**Authors:** Jie Sun, Qi Shen, Haiqi Lu, Zhinong Jiang, Wenxia Xu, Lifeng Feng, Ling Li, Xian Wang, Xiujun Cai, Hongchuan Jin

**Affiliations:** ^1^ Laboratory of Cancer Biology, Provincial Key Lab of Biotherapy in Zhejiang, Sir Runrun Shaw Hospital, Medical School of Zhejiang University, Hangzhou, China; ^2^ Department of Medical Oncology, Sir Runrun Shaw Hospital, Medical School of Zhejiang University, Hangzhou, China; ^3^ Department of Pathology, Sir Runrun Shaw Hospital, Medical School of Zhejiang University, Hangzhou, China; ^4^ Division of Hematopoietic Stem Cell and Leukemia Research, City of Hope National Medical Center, Duarte CA, USA; ^5^ Department of General Surgery, Sir Runrun Shaw Hospital, Medical School of Zhejiang University, Hangzhou, China

**Keywords:** Ras, TDG, oncogenesis, ING4

## Abstract

Ras is aberrantly activated in many cancers and active DNA demethylation plays a fundamental role to establish DNA methylation pattern which is of importance to cancer development. However, it was unknown whether and how Ras regulate DNA demethylation during carcinogenesis. Here we found that Ras downregulated thymine-DNA glycosylase (TDG), a DNA demethylation enzyme, by inhibiting the interaction of transcription activator ING4 with TDG promoter. TDG recruited histone lysine demethylase JMJD3 to the Fas promoter and activated its expression, thus restoring sensitivity to apoptosis. TDG suppressed *in vivo* tumorigenicity of xenograft pancreatic cancer. Thus, we speculate that reversing Ras-mediated ING4 inhibition to activate Fas expression is a potential therapeutic approach for Ras-driven cancers.

## INTRODUCTION

Pancreatic cancer is the fourth common cause of deaths due to cancer (http://globocan.iarc.fr). It has the worst 1 and 5 year survival rates of all cancers. Most of pancreatic cancer are sporadic and common risk factors include chronic pancreatitis, diabetes, cigarette smoking and heavy alcohol consumption. Strikingly, more than 90% pancreatic cancer have mutations in Ras oncogene [1]. Oncogenic point mutations leading to the constitutive activation of Ras oncoprotein occurs in 30% of human cancers [2, 3] while epigenetic down-regulation of inhibitors in Ras signaling pathway represent as alternative mechanisms for the activation of Ras signaling [4–7].

In addition to genetic mutations, epigenetic changes mainly deregulation of DNA methylation also contributes to the pathogenesis of pancreatic cancer [8, 9]. DNA methylation occurring within CpG dinucleotides of the promoter region usually function as transcriptional silencers to suppress gene expression. Oncogenic Ras signaling can promote DNA hypermethylation of tumor suppressor genes (TSGs) to facilitate cancer development by remodeling cell metabolism and many other biochemical processes [10–13]. Cytosine methylation catalyzed by DNA methyltransferases (DNMTs) has been well studied [14]. In contrast, the investigation of active DNA demethylation in mammalian somatic cells was still in its infancy [15, 16]. It was unknown whether and how Ras signaling regulates active DNA demethylation in human cancer cells.

In this study, we found that active Ras suppressed the transcription of thymine-DNA glycosylase (TDG) which encodes one of critical proteins to carry out active DNA demethylation [17, 18]. TDG can recruit histone lysine demethylase JMJD3 to Fas promoter and activated its expression to restore sensitivity to apoptosis. Consequently, TDG effectively suppressed *in vivo* tumorigenecity of xenograft pancreatic cancer. Therefore, reversing Ras-mediated ING4 suppression to activate TDG expression and subsequent Fas expression could a promising approach for the target therapy of pancreatic cancer and other Ras-driven cancers.

## RESULTS

### Ras inhibits TDG expression

**Figure 1:**
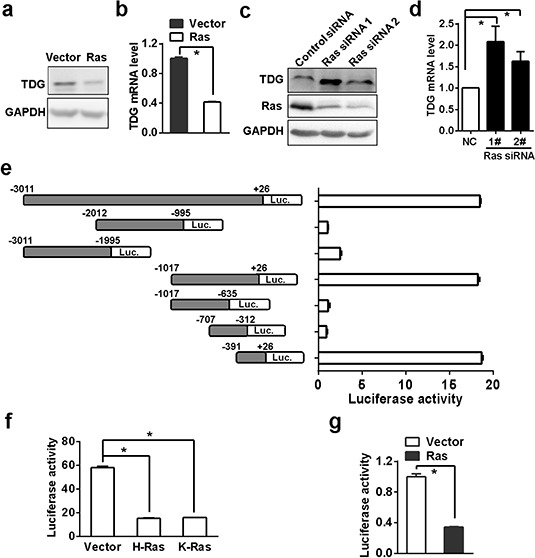
Ras inhibits TDG expression TDG expression in H-Ras transformed NIH3T3 cells was determined by Western blotting **a.** and qRT-PCR **b.** (*p* < 0.05, Student's *t* test). TDG expression after H-Ras depletion was determined by western blotting **c.** and qRT-PCR **d.** (*p* < 0.05, Student's *t* test). **e.** Luciferase expression driven by different length of TDG promoter regions were determined by measuring luciferase activities in HEK293T cells (*p* < 0.05, Student's *t* test). **f.** Luciferase expression driven by the −391/+26 region were determined by measuring luciferase activities in HEK293T cells transfected with either H-Ras^Q61L^ or K-Ras^G12V^ (*p* < 0.05, Student's *t* test). **g.** Luciferase expression driven by the −391/+26 region were determined by measuring luciferase activities in H-Ras transformed NIH3T3 cells (*p* < 0.05, Student's *t* test).

In order to clarify the relevance of Ras signaling to active DNA demethylation, we firstly analyzed the expression of the three most important enzymes involved in DNA demethylation, TET1 (ten-eleven translocation 1), AID (activation induced deaminase) and TDG, in Ras-transformed NIH3T3 cells. Interestingly, expression of the three enzymes in transformed cells were decreased (Figure 1a and 1b, and data not shown). In the current study we will focus on the regulation of TDG by Ras whereas the effect of Ras on other two enzymes will be reported elsewhere.

Then we determined TDG expression after H-Ras depletion in transformed cells. After treating with H-Ras siRNA for 72 hrs, either mRNA or protein of TDG was significantly increased (Figure 1c and 1d). It has been reported that TDG expression can be inhibited in multiple myeloma cell lines through promoter methylation [19], and indeed there is a CpG island around the first exon of mouse TDG gene. However, TDG mRNA did not increase after treating the transformed cells with DNMT inhibitor, 5-aza-2-deoxycytidine (Supplementary Figure 1), and TDG was actually not hypermethylated in both transformed and untransformed NIH 3T3 cells (Supplementary Figure 1). So we turned to search for alternative mechanisms responsible for Ras-mediated TDG downregulation.

By employing a series of luciferase reporter constructs driven by different length of DNA fragment cloned from the upstream of TDG transcription start site (TSS), we finally ascertained the −391/+26 region was crucial for TDG transcription (Figure 1e). The transcription activity of −391/+26 region was reduced in the presence of either active H-Ras or K-Ras (Figure 1f). Meanwhile, the transcription activity of −391/+26 region significantly lower in oncogenic Ras transformed NIH3T3 cells than in parental NIH3T3 cells (Figure 1g), confirming that oncogenic Ras signaling suppressed TDG expression at the transcription level.

### TDG is a target of ING4

**Figure 2:**
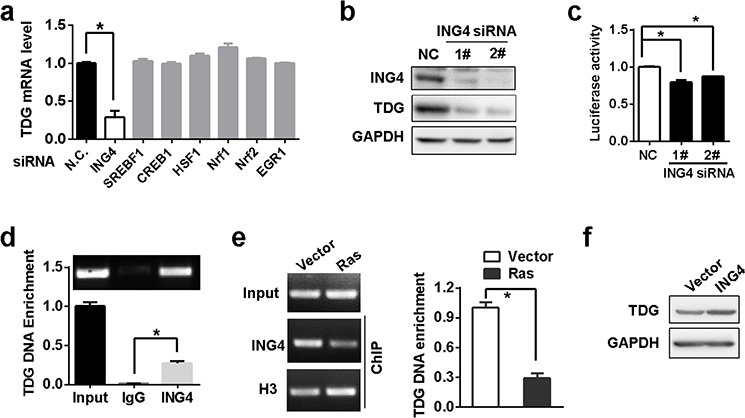
TDG is a target of ING4 **a.** TDG mRNA level after predicted transcription factors depletion was determined by qRT-PCR. The asterisk indicates significant difference (*p* < 0.05, Student's *t* test). **b.** TDG expression before and after ING4 depletion were determined by western blotting. **c.** Luciferase expression driven by the −391/+26 region were determined by measuring luciferase activities in HEK293T cells before and after ING4 depletion (*p* < 0.05, Student's *t* test). **d.** and **e.** Enrichment of TDG DNA by anti-ING4 antibody were determined by ChIP-qPCR (*p* < 0.05, Student's *t* test). **f.** TDG expression in Ras transformed NIH3T3 cells with or without ING4 overexpression were analyzed by Western blotting.

In order to identify transcription factors that activate TDG expression, we scanned transcription factor-binding sites within −391/+26 region (TRANSFAC scoring matrix, http://www.biobase-international.com/). Several candidate transcription factors were identified, such as Nrf1, Nrf2, SREBF1, CREB1, HSF1, EGR1 and ING4. However, the expression of TDG was reduced only in the presence of ING4 siRNA but not other siRNAs (Figure 2a and 2b). In addition, ING4 depletion inhibited the luciferase activity driven by the −391/+26 region (Figure 2c). Meanwhile, chromatin immunoprecipitation (ChIP) further confirmed the binding of ING4 to TDG promoter *in vivo* (Figure 2d). However, such interaction was abrogated in the presence of Ras, (Figure 2e), leading to the downregulation of TDG expression (Figure 1). As the consequence, the exogenous ING4 expression reverted Ras-mediated TDG repression in H-Ras transformed cells (Figure 2f).

### TDG is downregulated in pancreatic cancer

**Figure 3:**
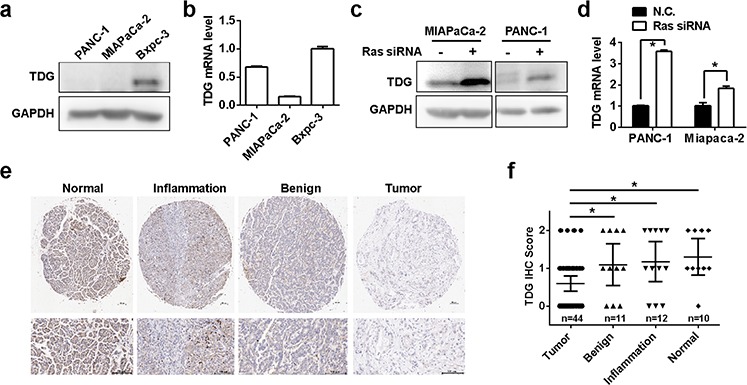
TDG is downregulated in pancreatic cancer TDG expression in human pancreatic cancer cell lines determined by Western blotting **a.** and qRT-PCR **b.** (*p* < 0.05, Student's *t* test). TDG expression in K-Ras mutated pancreatic cancer cell lines before and after K-Ras depletion were determined by Western blotting **c.** and qRT-PCR **d.** (*p* < 0.05, Student's *t* test). **e.** and **f.** TDG expression in different pancreatic tissues as indicated were determined by immunohistochemistry staining.

Oncogenic Ras mutations occur in more than 90% of pancreatic cancer of all grades [1], so we next examined the expression of TDG in human pancreatic cancer. As expected, TDG were down-regulated in K-Ras mutated pancreatic cancer cell lines Miapaca-2 and Panc-1, compared with the Bxpc-3 in which the K-Ras gene was wild type (Figure 3a and 3b). In addition, TDG expression in Miapaca-2 and Panc-1 cells were restored once K-Ras expression was knock-down by its siRNA (Figure 3c and 3d). We further analyzed TDG expression in various pancreatic tissues. Importantly, TDG expression in human pancreatic cancer tissues were significantly decreased when compared with its expression in normal pancreas or pancreas with chronic inflammation or benign tumors (Figure 3e and 3f). However, we failed to find any significant associations of TDG expression with clinico-pathological feature such as differentiation, gender (data not shown).

### TDG functions as a tumor suppressor by inducing apoptosis

**Figure 4:**
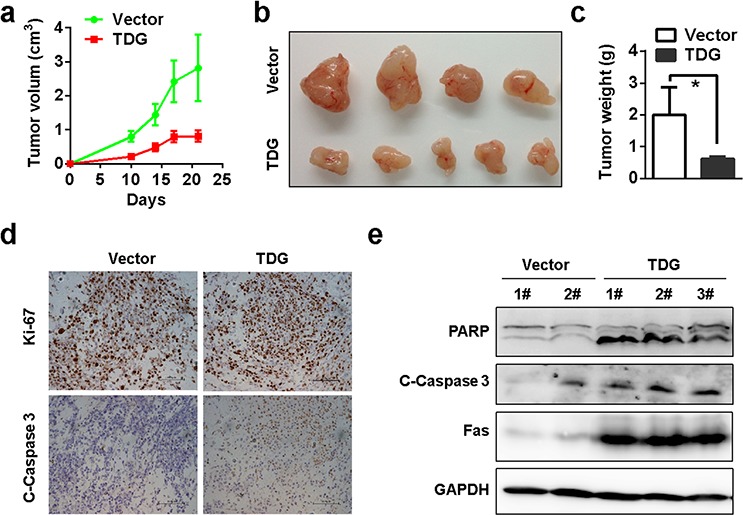
TDG functions as a tumor suppressor by inducing apoptosis *In vivo* growth of TDG overexpressed MIAPACA-2 cells was recorded as shown in **a. b.** and **c.** (*p* < 0.05, Student's *t* test). **d.** Expression of Ki-67 and cleaved Caspase-3 in xenograft tumors were determined by immunohistochemistry staining. **e.** Cleavage of PARP and Caspase-3 in xenograft tumors was determined by Western blotting.

Next, we explored the role of TDG as a tumor suppressor in pancreatic cancer. Unexpectedly, overexpression of TDG did not induce cell death or cell growth arrest *in vitro* (Supplementary Figure 2). However, the *in vivo* growth of tumors formed by pancreatic cancer cells with ectopic TDG expression was significantly retarded (Figure 4a 4b and 4c). Moreover, cleaved-Caspase3 and -PARP but not Ki-67 was increased in cells with ectopic TDG expression (Figure 4d and 4e), indicating that ectopic TDG expression induced cell apoptosis but not proliferation inhibition.

### TDG activates Fas transcription to promote apoptosis

**Figure 5:**
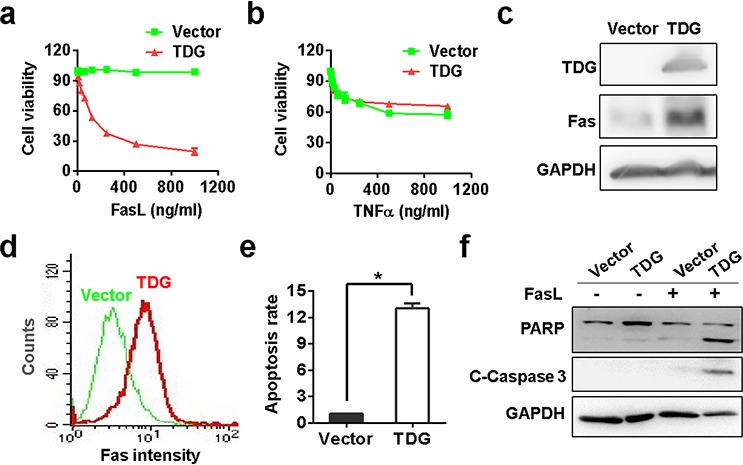
TDG activates Fas transcription to promote apoptosis Viabilities of TNFα **a.** or FasL **b.** treated MIAPACA-2 cells with or without TDG overexpression were determined by MTS assay (*p* < 0.05, Student's *t* test). Fas expression in MIAPACA-2 cells with or without TDG overexpression were determined by Western blotting **c.** and FCM **d.** (*p* < 0.05, Student's *t* test). **e.** FasL induced apoptosis of MIAPACA-2 cells with or without TDG overexpression were determined by FCM (*p* < 0.05, Student's *t* test). **f.** Cleavage of PARP and Caspase-3 in FasL treated MIAPACA-2 cells with or without TDG overexpression were determined by Western blotting.

The different effect of TDG *in vitro* and *in vivo* made us infer that TDG promoted apoptosis through the extrinsic pathway. To define the exact signaling pathway regulating apoptosis, we treated the TDG overexpression cells with TNFα and FasL, two major inducers of extrinsic apoptosis. As shown in Figure 5a and 5b, FasL but not TNFα inhibited the viability of TDG overexpressed cells in a concentration dependent manner, indicating that TDG might promote the expression of Fas. Indeed, both Western blotting and flow cytometry assay confirmed the increase of Fas protein in TDG overexpressing cells (Figure 5c and 5d) or tumors formed by TDG overexpressing cells (Figure 4e). Consistently, FasL induced apoptosis of pancreatic cancer cells only in the presence but not absence of TDG expression (Figure 5e and 5f).

### TDG recruits JMJD3 to activate Fas transcription

**Figure 6:**
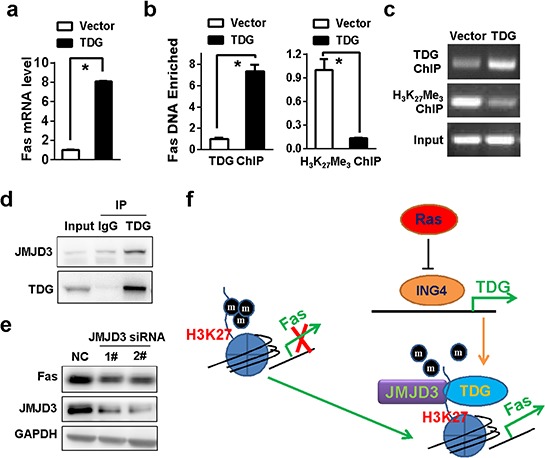
TDG recruits JMJD3 to activate Fas transcription **a.** Fas mRNA level in MIAPACA-2 cells with or without TDG overexpression were determined by qRT-PCR (*p* < 0.05, Student's *t* test). **b.** and **c.** Enrichment of Fas DNA by anti-TDG or anti-H_3_K_27_ Me_3_ antibody was determined by ChIP-qPCR (*p* < 0.05, Student's *t* test) and ChIP-PCR, respectively. **d.** The interaction of TDG and JMJD3 was determined by co-IP. **e.** Fas expression before and after JMJD3 depletion in TDG overexpressed MIAPACA-2 cells were determined by Western blotting. **f.** A proposed working model: TDG was transcriptionally repressed by Ras-mediated inhibition of ING4. JMJD3 was recruited by TDG to Fas promoter and activated Fas expression by demethylating H_3_K_27_.

The role of TDG in promoting active DNA demethylation indicated that TDG may promote Fas expression though activating Fas promoter demethylation. As expected, Fas mRNA level was significantly raised in TDG overexpressing cells (Figure 6a). However, promoter hypermethylation seems to be not relevant to the regulation of Fas expression. 5-aza-2-deoxycytidine failed to increase Fas mRNA (Supplementary Figure 3) and Fas promoter was actually not hypermethylated in Miapaca-2 cells (Supplementary Figure 3). In addition to its roles in active DNA demethylation, TDG also implicated in transcriptional regulation. Indeed, TDG bound to the promoter of Fas gene (Figure 6b and 6c). Meanwhile, the level of H_3_K_27_ trimethylation in Fas promoter was markedly reduced in the presence of TDG expression (Figure 6b and 6c). Interestingly, TDG could interact with JMJD3, the major histone demethylase of trimethylated H_3_K_27_ (Figure 6d), and Fas expression were reduced once JMJD3 was depleted (Figure 6e), indicating that TDG recruit JMJD3 to facilitate histone H3K27 demethylation and subsequent Fas transcription (Figure 6f).

## DISCUSSION

In consideration of its role in DNA repair and demethylation, TDG may play as a tumor suppressor. Indeed, loss of TDG function led to the mutator phenotype and increased susceptibility to carcinogenesis [20, 21]. Such loss of TDG function was often attributed to genetic changes. For example, human TDG actually locates at chromosome 12q22-q24.1, one of regions frequently lost in many human cancers including gastric cancer and pancreatic cancer [20, 22]. Meanwhile, germline mutations in TDG were also detected in patients with familial colorectal cancer [21]. On the other hand, TDG expression in human cancers can also be regulated by epigenetic changes. For instance, the expression of TDG could be targeted by miR-29 family [23, 24]. In addition, TDG promoter can be hypermethylated, leading to the silencing of its expression [19, 25]. However, we found oncogenic Ras repressed TDG transcription independent of promoter hypermethylation (Supplementary Figure 1). Furthermore, we identified a novel mechanism resulting in the loss of TDG function (Figure 6). Certainly, we could not exclude other transcription factors important to activate TDG transcription. It has been reported that TDG is actually a target of p53 [26]. However, we found no binding sites for p53 in the minimal promoter region we identified, indicating that p53 may regulate TDG transcription through other DNA elements or in a cell-specific manner.

ING4, the newly identified transcription activator of TDG, is a well-defined tumor suppressor in many types of cancers [27, 28]. In consistence with its function to activate TDG transcription, ING4 was found to be crucial for gene transcription via acetylation of chromatin substrates as a consequence of its association with histone acetyltransferase complex [29]. However, how oncogenic Ras signaling regulation ING4 function remains further investigations.

Ectopic TDG expression led to the activation of extrinsic apoptosis through the induction of Fas expression. Interestingly, oncogenic Ras signaling has been shown to inhibit apoptosis by regulating the expression of various players in the apoptosis response [30–32]. As the major death receptor, Fas was downregulated in many cancer cells. The predominant mechanism of its downregulation is promoter hypermethylation [12]. Much to our surprise, TDG seems to activate Fas transcription independent of DNA demethylation although it indeed bound to Fas promoter. Firstly, pharmacological demethylation failed to increase Fas mRNA. Secondly, bisulfite gene sequencing revealed that Fas promoter was actually not hypermethylated in pancreatic cancer cells. Instead, we found TDG activated Fas expression through epigenetic chromatin remodeling. It recruited JMJD3 to Fas promoter and promoted the demethylation of lysine 27 in Histone 3, which is a well-known marker for active gene transcription. Interestingly, TDG could interact with the transcription-activating histone acetyltransferase (CBP/p300) [33–36], which protected gene promoters from polycomb repressive complex (PRC) -mediated H_3_K_27_ trimethylation [37]. However, the transient transfection of TDG also led to the activation of Fas transcription, strongly supporting an active demethylation of H_3_K_27_.

In conclusion, oncogenic Ras suppressed the transcription of TDG by inhibiting the interaction of its transcription activator ING4 with TDG promoter. TDG functions as a tumor suppressor by promoting the demethylation of H_3_K_27_ in Fas promoter and activating Fas expression. Therefore, reversing Ras-mediated downregulation of TDG expression and subsequent Fas expression could a promising approach for the target therapy of pancreatic cancer and other Ras-driven cancers.

## MATERIALS AND METHODS

### Cells, antibodies, chemicals and plasmids

All cell lines (Bxpc-3, MIAPACA-2, PANC-1 and NIH3T3) were purchased from American Type Culture Collection (ATCC, Manassas, VA, USA). Except for the Bxpc-3, which was cultured in RPMI 1640 medium (Invitrogen, Carsbad, CA, USA), all other cell lines were incubated in DMEM medium (Invitrogen) supplemented with 10% fetal bovine serum at 37°C with 5% CO_2_ and 95% humidity. Antibodies for ING4 and JMJD3 were bought from Abcam. Anti-TDG antibody was from Santa Cruz and anti-Fas, GAPDH, tri-methyl-H_3_K_27_, cleaved-Caspase3 and PARP were from Cell Signaling Technology (Boston, MA, USA). All chemical inhibitors were bought from Sigma. K-Ras (1-688bp) full length open reading frame (ORF) was amplified by PCR using the GoTaq Green Master Mix (M7123, Promega, Madison, WI, USA) with cDNA reversing transcription from total RNA of PNAC-1 cells. The primers used were listed in Table 1. The PCR products were cloned into the pGEM-T Easy Vector (Promega, Madison, WI, USA). After sequence verification, the inserts were sub-cloned using BamHI and XhoI restriction sites into a mammalian expression vector pCMV-3Tag-7 (Agilent, La Jolla, CA, USA).

**Table 1:** Primers used in the study

Primer name	Primer sequence
K-Ras-cloning	F: GGATGACTGAATATAAACTTGTGGTAG R: GGTTACATAATTACACACTTTGTCTTTG
hTDG-RT-PCR	F: AAAATCTGGCAAGTCTGC R: GGTCCAGGGTAATGATGC
mTDG-RT-PCR	F: CGCAAGAGGACGCAAAGA R: TGCCCATTCGGAACATCG
hTDG-promoter	F1: GGGGTACCTGCAGGAGCAGTCTTGGA R1: GGGCTAGCTCCTCGGAGCCAAATCC F2: GTGGTACCGCAACCTTGCGAATCTC R2: GTGCTAGCTGAGATTCGCAAGGTTGC F3: GTGGTACCATGTGCCAGGTTCTGAGT R3: GTGCTAGCACACTACTCAGAACCTGG F4: CTGGTACCTCATCCTGCAAACTAGAA R4: CTGCTAGCCAGCTGGCTTTTGTTTCA F5: CTGGTACCTTCACCCTCATTTCACAGAT R5: CTGCTAGCTTGAAAGTGGAAAACCTG
hTDG-Chip-PCR	F: CAAAGACCCTCCCTCACA R: TAGGACAGCCCAATCACG
mTDG-Chip-PCR	F: AGCCCTACTCCTCATCACA R: GACAGTGGCAGGCAGAAC
hFas-Chip-PCR	F: GCATCTGGACCCTCCTACCTC R: CGCATCAAGGCCCAAGAAA

TDG (1-1233bp) and ING4 (1-747bp) full length ORF inserted into neomycin resistant mammalian expression vector EX-Z4461-M14 was purchased from GeneCopoeia (Rockville, MD, USA). H-Ras (Q61L) cDNA was purchased from Upstate (Lake Placid, NY, USA).

### siRNA or plasmid transfection

siRNAs were synthesized by GenePharma (Shanghai, China). The sequences of siRNAs were listed in Table 2. For siRNA transfection, Cells were seeded overnight in 6-well plates (2–3 × 10^5^/well) and transfected with siRNA duplexes (10 nM) using Lipofectamine™ RNAiMAX transfection reagent (Invitrogen) according to the standard protocol. siRNA duplex with a scrambled sequence that will not target any specific mRNA was used as a negative control. Cells were harvested for RNA and protein extraction after 72 hr. Plasmids were transfected similarly except that FuGENE HD (Roche Applied Science, Mannheim, Germany) were applied according to the standard protocol provided.

**Table 2:** siRNAs used in the study

siRNA name	target sequence
H-Ras	1#: GGAAGCAGGTGGTCATTGA
	2#: CCAGCTGATCCAGAACCAT
K-Ras	1#: GAGGAGTACAGTGCAATGA
	2#: GCTCAGGACTTAGCAAGAA
ING4	1#: GCCACTGAGTATATGAGTA
	2#: GCTTGCCATGCAGACCTAT
JMJD3	1#: GCGATGTGGAGGTGTTTAA
	2#: GTGACAAGGAGACCTTTAT
CREB1	GCCACAGATTGCCACATTA
HSF1	CCTGAAGAGTGAAGACATA
NRF1	CCGTTGCCCAAGTGAATTA
NRF2	GCCCATTGATGTTTCTGAT
EGR1	CCCGGTTACTACCTCTTAT

### Western blotting

Cells were scraped and lysed in Cytobuster Protein Extraction Reagent (Novagen, Darmstadt, Germany) and protein concentrations were determined by Bio-Rad protein assay kit II (Bio-Rad Laboratories, Hercules, CA, USA). Equal amounts of cellular protein were resolved by SDS-PAGE and transferred to PVDF membrane. Proteins of interest were detected with the indicated primary antibodies followed by suitable HRP-conjugated second antibodies and autoradiographed with enhanced chemiluminescence (Millipore, Billerica, MA, USA).

### RNA Extraction and quantitative real-time RT-PCR

Total RNA was extracted using Trizol reagent (Invitrogen) according to the manufacturer's instructions. RNA concentrations were quantified by NanoDrop 1000 (Nanodrop, Wilmington, DE, USA). Reverse transcription reaction was performed using 1 μg of total RNA with High Capacity cDNA Reverse Transcription kit (Applied Biosystems, Foster City, CA, USA). The mRNA level was determined by quantitative real-time PCR using SYBR Green Master Mix Kit and ABI 7500 Real-Time PCR System (Applied Biosystems). Human glyceraldehyde-3-phosphate dehydrogenase (GAPDH) was used as an internal control of RNA integrity. Primers used were listed in Table 1.

### Luciferase activity assay

DNA fragments cloned from upstream of TDG TSS were inserted into pGEM-T Easy Vector for sequencing. The primers used were list in Table 1. pGL2 vector containing correct insert was co-transfected with pRL-TK into cells (1 × 10^5^) seeded in 12-well plates. 48 hr later, the activities of firefly luciferase and renilla luciferase were measured using the Dual-GloTM luciferase assay system (E2940, Promega) according to the manufacturer's instructions. Relative luciferase activity was normalized with renilla luciferase activity.

### Flowcytometry analysis

To determine the expression of Fas protein, cells transfected with TDG overexpression vector were washed twice with cold 1 × PBS and then resuspended in 100 μl staining buffer containing 5 μl anti-human CD95 (APO-1/Fas) FITC monoclonal antibody (11–0959, eBioscience, San Diego, CA, USA). And 30 minutes later, another 400 μl staining buffer was added into the suspension and cells were tested by FCM analysis.

Cell apoptosis was determined by using the FITC Annexin V Apoptosis Detection Kit I (BD Bioscience, Bedford, MA, USA) according to the manufacturer's instructions. Cells were washed twice with cold 1 × PBS and then resuspended in 1× Binding Buffer at a concentration of 1 × 10^6^ cells/ml. Then mixed 100 μl of cells with 5 μl of FITC Annexin V and 5 μl PI, gently vortexed and incubated for 15 min at room temperature in darkness before analyzing by flow cytometry.

### Immunoprecipitation

Cells were lysed in 1 ml of lysis buffer (20 mM Tris-HCl, pH 7.5, 1% Nonidet P-40 and 10% glycerol) containing protease inhibitors (Complete EDTA-free Protease Inhibitor Cocktail Tablets, Roche). Primary antibodies were added into the pre-cleared cell lysates and incubated overnight at 4°C with gentle agitation. The immunocomplexes were precipitated by Protein G magnetic beads. The isolated beads were resuspended in 1 × SDS-PAGE loading buffer and protein levels were analyzed by Western blot.

### Chromatin immunoprecipitation (ChIP)

ChIP assay was performed by using SimpleChIP™ Enzymatic Chromatin IP Kit (9003, Cell Signaling Technology) according to the manufacturer's instructions. In brief, cells were fixed and lysed, and then chromatin was harvested and fragmented using sonication. Antibodie specific to ING4, Flag or H_3_K_27_Me_3_ was used to recruit the target DNA and the complex was precipitated by Protein G magnetic beads. After immunoprecipitation, the protein-DNA complex was reversed and the DNA was purified. The enriched DNA was subjected to PCR analysis. The primers used were listed in Table 1.

### Cell viability assay

Cells viability was determined by CellTiter 96^®^ AQueous Non-Radioactive Cell Proliferation Assay kit (Promega). In brief, the cell suspension was seeded in 96-well plate at a density of 3 × 10^3^ cells/well for 24 hr and incubated overnight. MTS was added and the absorbance at 490 nm of each well was measured 72 hr later directly.

### Tissues microarray and immunohistochemistry

Immunohistochemistry (IHC) was performed as previously described [5]. Pancreatic cancer tissue microarray was purchased from US Biomax (PA2081, Rockville, MD, USA), containing 44 cases of pancreatic adenocarcinoma, 11 cases of benign pancreatic tumor, 12 cases of chronic pancreatitis, and 10 normal pancreatic tissue. After IHC staining, all specimens were strictly evaluated by two senior pathologists. Staining intensity was assessed as none (0), weak (1), medium (2) and strong (3).

### *In vivo* tumor growth

For tumor growth assay, cancer cells were injected subcutaneously into the flank of nude mice (Shanghai Lab. Animal Research Center, Shanghai, China). The size of tumor was recorded every 3 days. Mice were sacrificed 21 days after cell inoculation and the tumors were removed and weighted. Tumor tissues were immediately snapped frozen in liquid nitrogen and stored at −80°C for protein extraction, or fixed in 10% formaldehyde for IHC.

## SUPPLEMENTARY FIGURES


