# Supplementary material for: Oncogenic Ras suppresses ING4-TDG-Fas axis to promote apoptosis resistance
Source: Oncotarget. 2015 Oct 24;6(39):41997–2007. doi: 10.18632/oncotarget.6015 (PMC4747204; doi:10.18632/oncotarget.6015)
Supplement: Supplementary file 1 [file oncotarget-06-41997-s001.pdf]

SUPPLEMENTARY FIGURES

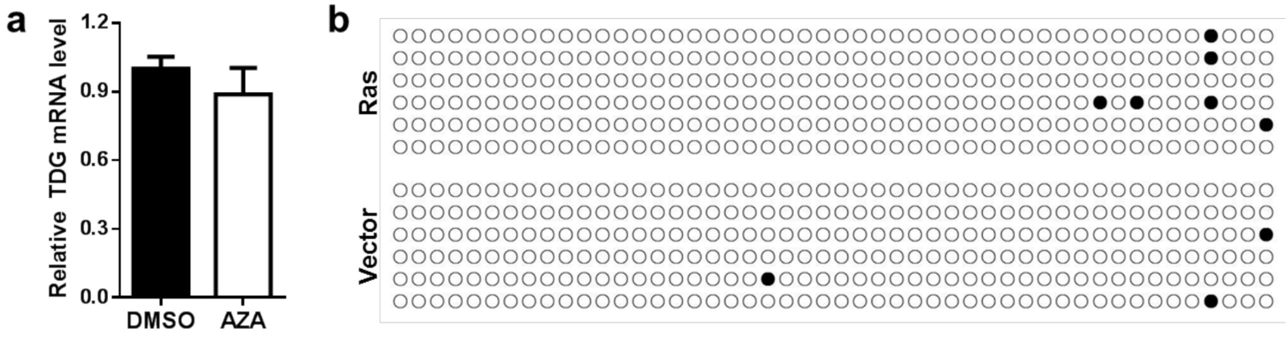

Supplementary Figure S1: a. TDG mRNA expression in NIH3T3-Ras cells before and after 5-Aza-2'-deoxycitydine (Aza) treatment were determined by real time RT-PCR (*p* > 0.05). b. The methylation status of TDG promoter in NIH3T3 cells with (Ras) or without Ras (Vector) transfection were analyzed by bisulfite genome sequencing.

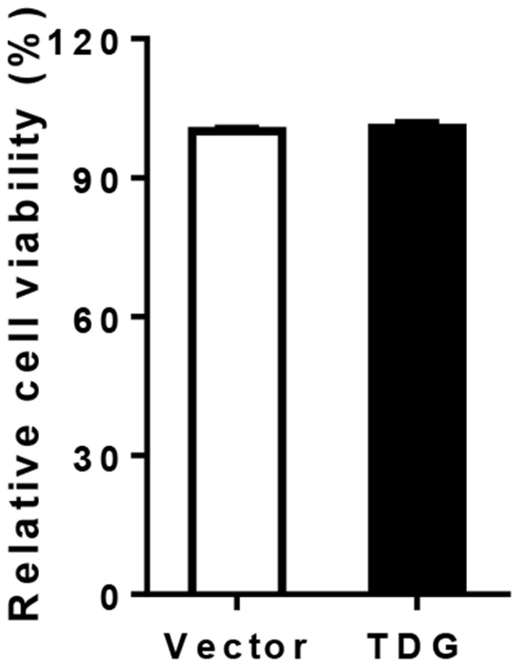

Supplementary Figure S2: The viability of MIAPACA-2 cells transfected with or without TDG were analyzed by MTS assay (p > 0.05).

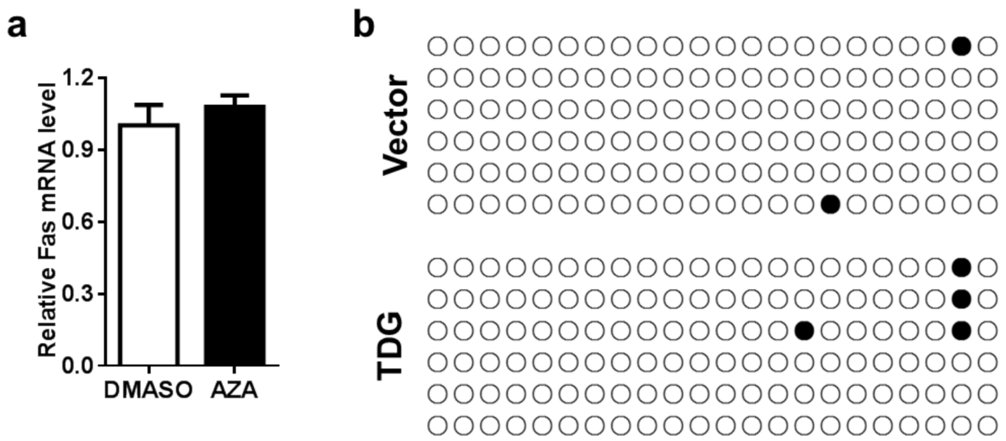

Supplementary Figure S3: a. Fas mRNA expression in MIAPACA-2 cells treated with or without Aza were determined by real time RT-PCR ($p > 0.05$). **b.** The methylation status of Fas promoter in MIAPACA-2 cells with (TDG) or without TDG (Vector) transfection were analyzed by bisulfite genome sequencing.
